# Landscape configurations of refuge areas that delay the evolution of resistance to Bt sugarcane: an agent based modeling approach

**DOI:** 10.1093/jee/toad104

**Published:** 2023-07-01

**Authors:** Dirk Johannes Human, Linke Potgieter

**Affiliations:** Department of Logistics, Faculty of Economics and Business Management, Stellenbosch University, Stellenbosch, South Africa; Department of Logistics, Faculty of Economics and Business Management, Stellenbosch University, Stellenbosch, South Africa

**Keywords:** Bt sugarcane, *Eldana saccharina* Walker, resistance management, refuge planning, agent-based modeling

## Abstract

Although transgenic crops expressing genes from the bacterium *Bacillus thuringiensis* (Bt) are considered to be an effective pest control method, reckless usage adds environmental pressure on a pest population to develop resistance to the protein over time. The use of small portions of non-Bt crop (refuge areas) limits the rate of resistance development. Strains of Bt sugarcane for the South African market are being developed, and a prerequisite to releasing such a product on the market is a recommendation on the size and layout of the refuge areas. In this article, an agent-based simulation model is used to test the effectiveness of different landscape configurations of refuge areas in Bt sugarcane against resistance development occurring in an associated lepidopteran pest population. Individual insects are modeled as agents on an underlying sugarcane field that can either be Bt or refugium. The model is applied to 2 hypothetical case studies, each focusing on a specific aspect of refugia planning. The first focuses on the size and distribution of refuge, and the second on the shape of the refuge. A conservative general recommendation of 30% per farm, planted in large blocks on farms, is made based on simulation results and what is currently known about the target pest species, to provide regulatory bodies as well as growers with a starting point on how to regulate and plan refuge areas in South African Bt sugarcane.

## Introduction

The South African Sugarcane Research Institute (SASRI) is in the process of developing transgenic sugarcane strains that contain genes from the bacterium *Bacillus thuringiensis* (Bt), allowing the plant to express the insecticidal proteins that are deadly to the lepidopteran pest species *Eldana saccharina* Walker. *Eldana saccharina* is a significant pest species in sugarcane in South Africa, with temperature playing a fundamental role in its development (Atkinson and Carnegie 1989, Kleynhans et al. 2018). SASRI, are required to provide refuge requirements to the South African Department of Agriculture, Forestry and Fisheries before releasing the new strains to the market. These requirements are not necessarily the same for each Bt crop, and should be uniquely determined.

The first instances of transgenic crops containing Bt genes were tested in the early 1990s (Koziel et al. 1993) and Bt maize, the first commercially produced Bt crop, was first registered with the EPA in 1995 (Aroian 2018). Bt crops have since been widely embraced and readily planted. Although significant pest suppression has been reported, it was found that some pests were displaying a level of resistance to the Bt crops. Studies on 2 moths, *Heliothis virescens* and *Ostrinia nubilalis* Hübner, showed that the frequency of resistant alleles to be between 0.01% and 0.1% before the introduction of Bt crops (Gould et al. 1992, Bourguet et al. 2003). A study by Tabashnik et al. (2003) indicated no increase in the frequency of resistant alleles since the introduction of Bt crops, however, there are several cases where field resistance has been reported in Bt crop (Luttrell et al. 2004, Van Rensburg 2007, Gassmann et al. 2011).

As a result of resistance development, regulatory bodies from every country where Bt crops are planted require that measures to curb insect resistance development are put in place to manage the efficacy of the Bt crops (Aroian 2018). The long timelines between the development of new strains also require the measures to curb resistance for several years, with Cristofoletti et al. (2018) specifying at least 10 yr. The primary mitigation measure is the use of a High-Dose/Refuge strategy (Reynolds and Martinez 2017). This approach uses Bt crops with very high doses of the pesticidal proteins combined with refuge areas containing non-Bt crops that can house populations of susceptible insects that could mate with resistant insects emerging from the Bt crops to help dilute the resistance gene in the population, thereby curbing resistance development. (Ostlie et al. 1997) describes 3 assumptions that need to hold for the strategy to be successful: (i) Resistance genes must be sufficiently rare, (ii) Resistance genes must be nearly recessive, meaning that heterozygote individuals should have a similar mortality rate to the Cry proteins as a susceptible individual, and (iii) refuge areas should be able to maintain populations of susceptible individuals that are able to come into contact with resistant individuals within their typical dispersal area.

General recommendations for refuge areas vary according to the regulatory body of the particular country as well as the producer of the crop. In the United States, for example, the EPA has requirements varying between 5% and 50% depending on how many Cry proteins the crop contains and the pest it is targeting (Reynolds and Martinez 2017). Most Bt crops currently available on the market and under development contain multiple Cry proteins, ensuring that each plant has more than 1 “mode of action” (MOA) to kill the target pest (Cristofoletti et al. 2018).

In this paper, the problem of determining the best landscape configuration of refuge areas within a Bt sugarcane environment to combat resistance development in the target pest, *E. saccharina*, is considered using agent-based simulation modeling. Bt sugarcane containing 2 modes of action are assumed in this study. Related studies such as those by Tabashnik et al. (1990, 2003) and Butterfield (2002) consider a similar research question, namely the required size of refuge areas, but the models developed are population-based models that make spatial assumptions that may not be applicable in the typical *E. saccharina* on sugarcane scenario, and they do not address the question of spatial layout specifications. Other notable population-based modeling studies pertaining to resistance development in Bt crops include the study by (Roush 1998), where the “pyramiding” of different Cry proteins were found to reduce refuge requirements from 40% to 50% to as low as 10%, and the study by Cristofoletti et al. (2018) where the combination of protein efficacy, number of modes of action and refuge size were considered that could curb resistance development in the stalk corer *Diatraea saccharalis*. Spatially explicit population-based models have also been developed, although they are generally focused on a regional scale, with individual points in space being upwards of 4 hectares in size (Caprio and Tabashnik 1992, Caprio 1998, Peck et al. 1999, Storer et al. 2003, Sisterson et al. 2004, 2005, Tyutyunov et al. 2008).

Although the studies mentioned above all aim to simulate resistance development in a Bt crop scenario, most are focused on size requirements of refuge areas, with limited consideration of spatial layout requirements of refuge areas. Our study aims to address the question of both size and layout requirements of refuge areas in sugarcane and to capture genetic flow within a population more accurately by modeling individual insects with their own coded genome which can be passed from the parents to their offspring. The agent-based model developed in this study considers resistance development on a larger scale than the agent-based model developed by Garcia et al. (2016), where the impact of small scale larval movement on resistance development were considered. Practical and current planting practices are also considered. For example, the strip layout refugia commonly seen in maize fields would not be suitable for sugarcane, as sugarcane is generally planted in blocks. The objective is to provide decision support to the regulatory body of South Africa for determining the refuge requirements (size and layout prescription) for South African Bt sugarcane containing 2 modes of action (MOA) that should be adhered to by farmers, and also as a general guide to farmers on how to plan their refuge areas (Meyer and Snyman 2013, Snyman et al. 2018).

## Materials and Methods

### Choice of Methodology

To observe gene flow within a population more accurately, without making population-based assumptions, it was required that moths be represented in a model as individuals that can interact with each other and their environment. For this reason, it was decided that the most applicable simulation paradigm would be agent-based modeling. Significant groundwork for modeling *E. saccharina* population growth and dispersal as an agent-based simulation already exists in the work of van Vuuren et al. (2018), which further reinforces the choice of modeling paradigm.

For the research question considered in this study, it was necessary to model over multiple generations, crop cycles, and hectares. Given that 1 mated female *E. saccharina* can lay up to 500 eggs, a simulation of multiple generations over a large spatial area can quickly result in millions of agents in the system. In the work by Van Vuuren et al. (2018), the research focus was to investigate the impact of small scale behavior and interactions on the population dynamics of *E. saccharina*, resulting in a very detailed modeling of the biology, movement, and behavior of the insect. Whereas the model improved our understanding of the system’s emergent behavior, and what parameters contribute the most to these emergent patterns, the computational effort in running such a detailed model on a larger spatial and time scale was deemed to be infeasible for the purpose of our study. A higher level of abstraction was therefore required, and alternative software to allow for high performance computing. These adjustments are described in more detail in (Human 2019).

### Model Components

There are several design components to an agent-based simulation model, as described by Macal and North (2010). In the model developed for this study, the real-world system of *E. saccharina* on sugarcane are represented as different *agent types*, each type with their own *attributes*, such as age and gender, and *behaviors* such as oviposition, maturation, and movement, and the agents’ *environment*, describing the conditions and habitats surrounding the agents as they act and interact within the model, such as the interaction topology (in this case a 2-layer Euclidean-based environment), daily temperatures and time that passes. Finally, there are *interactions* that occur, such as the moths mating or seeking out favorable spots of sugarcane for oviposition.

The model contains 4 different agent types, namely *Eggs*, *Larvae* (which include pupae), *Adult moths*, and *Field*, each with an associated set of attributes, namely $\;E_{a}$, $L_{a}$, $A_{a}$, and $F_{a}$, and an associated set of methods, namely $E_{m}$, $L_{m}$, $A_{m}$, and $F_{m}$. The sets of attributes associated with each agent type, are *E*_*a*_ = {*Age, Female Gene*, *Male Gene*, *Number*, *Survival Rate}*, *L*_*a*_ = {*Age*, *Female Gene*, *Male Gene*, *Number*, *Stage 2 Indicator*}, *A*_*a*_ = {*Age, Gender, Fertility, Bt Resistance 1, Bt Resistance 2, Times Mated*}, and *F*_*a*_ = {*Age, Variety, Height, Stalk Capacity, Moth Capacity, Growth Rate, Stressed Rating*}, respectively. The sets of methods associated with each agent type, are *E*_*m*_ = {*Set Age*}, *L*_*m*_ = {*Set Age*}, *A*_*m*_ = {*Set Age*, *Set Fertility*, *Lay Eggs*}, and $F_{m}=${*Set Age, Set Capacity, Set Growth Rate, Set Height, Harvest*}. A detailed discussion of how the attributes and methods are implemented in the model, is provided in (Human 2019).

A Euclidean based simulation environment representing 25 ha with 2 different layers were used in the model, with a reflective hard boundary. The first level represents the ground and contains the field, egg, and larva agents as well as the fertile female agents in the process of laying eggs. For the purposes of our research, each cell in the ground layer represents 100 m^2^. The second level represents the canopy of the sugarcane and the sky where adult moth agents interact, move, and mate. Each cell in the sky layer represents an area of 2,500 m^2^, and sitting atop 25 (5 × 5) ground cells. The larger sky cells facilitate far flying in a more computationally efficient way.

The only climatic factor considered in this research was temperature, as it has a large seasonal impact on both *E. saccharina* and sugarcane growth. Temperatures used in the simulations were 730 days of base temperatures according to a local weather station in Kwazulu-Natal, South Africa. Other climatic factors were not explicitly included as parameters in the model, but were assumed to be normal, average conditions. Extreme conditions such as drought were not considered in the scope of this research.

### Base Model Logic

**Fig. 1.**
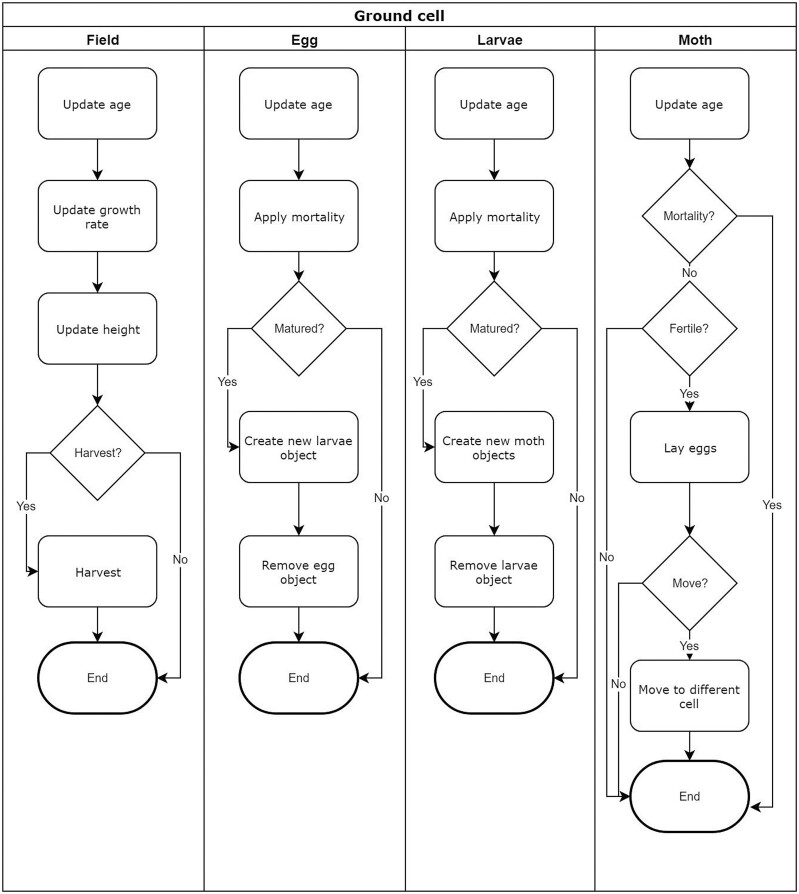
The process followed to update agents in the ground cells. Each agent type is updated independently, and simultaneously, to the other agent types.

**Fig. 2.**
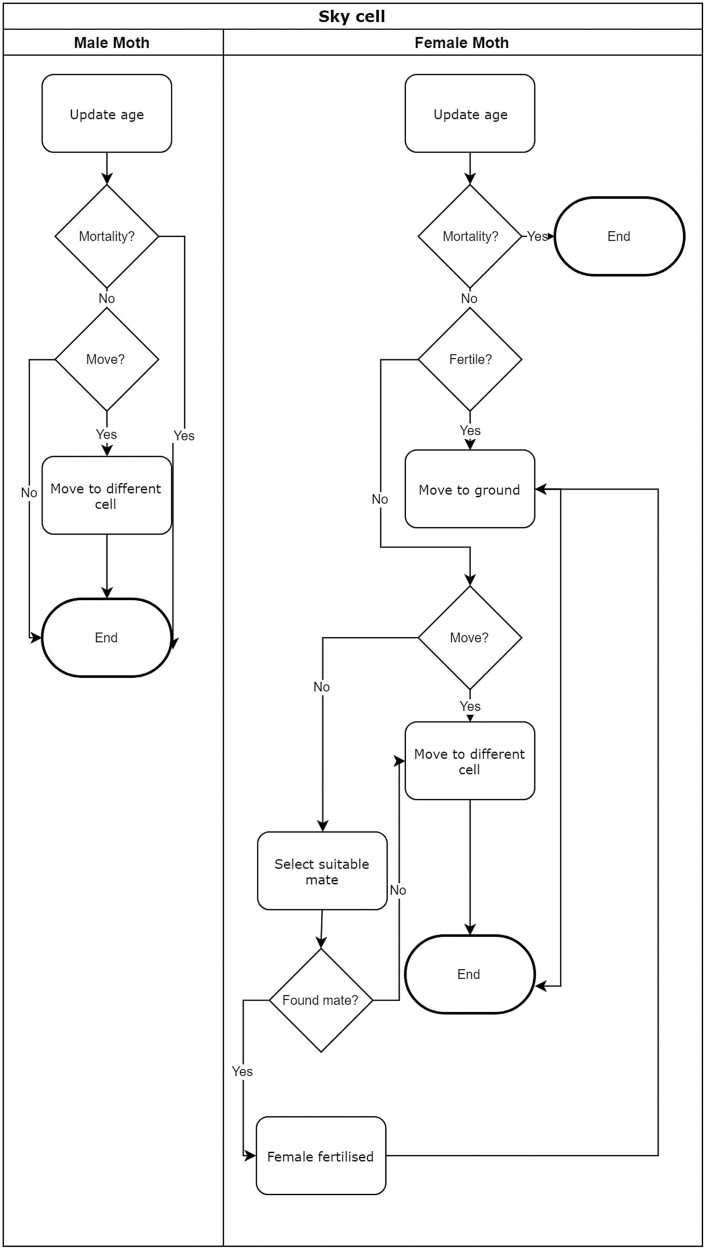
The process followed to update moth agents in a sky cell. All agents are updated simultaneously, and independently, from other agents.

In the model, time progresses in discrete daily time steps. The process followed to update all agents in the ground cell is detailed in Fig. 1. The sky cell can only be populated by adult moth agents and the process followed to update the moth agents is detailed in Fig. 2.

Moths are free to move within the simulated space, with 2 density-dependent biases included, namely the bias for female moths to move towards areas with higher moth densities, which would account for the female’s natural tendency to follow male pheromone concentrations, and the bias for male moths to seek out older, and thereby taller, sugarcane (Conlong et al. 2007, Berry et al. 2010). These biases would then ideally focus population concentrations around areas with more mature sugarcane that would be able to support larger populations of moths. For immature life stages, the sugarcane itself has a limit to the population of larvae it can support, ensuring that over infested areas of sugarcane would have a natural cap of insects it can support.

Due to computational effort, it was deemed infeasible to include the lek mating of *E. saccharina* as in the Van Vuuren et al. (2014) model. The lek system is mostly excluded, except that if a cell does not have enough males to form a lek (6 males), a female will move from her current cell to a neighboring cell in search of potential mates. A female moth randomly selects a male that has not already mated 5 times within a cell (Walton and Conlong 2016). The male moths that have already mated 5 times are not removed from the model once they reach this limit, as they should still be able to influence the female moth’s biased flight. Once the female has mated with a male in the sky cells, she will descend into the ground cells and start ovipositing eggs, with choice of ground cell a biased selection according to the age of sugarcane in the ground cells underneath the sky cell. Once a female enters a ground cell, she starts ovipositing, with movement between ground cells influenced by the available stalk capacity. A female that mates with a male would be set as fertilized with between 300 and 350 eggs (Walton and Conlong 2016), drawn from a uniform distribution, that are oviposited between 30 and 150 in a day for up to 5 days, with most eggs (50%) oviposited on the second day after emergence.

### Software

The model was developed using Python 3.6 with statically typed variables, which was compiled using the Cython 0.24 compiler to aid in performance. Early verifications and validations were performed on a 64-bit personal desktop computer with an Intel Core i7-6700 CPU running at 3.40GHz with 8GB of DDR3 memory. Full simulation runs were performed on the Lengau cluster, a super computer hosted by the Centre for High Performance Computing of the CSIR (http://www.chpc.ac.za).

### Verification and Validation

The model was verified incrementally during implementation, using typical techniques described in (Law and Kelton 2007). Model validation was done through face validation and consulting with entomological researchers, and through comparison with the population-based models developed by Potgieter et al. (2012, 2013) and the agent-based model developed by Van Vuuren et al. (2018) for *E. saccharina* population dynamics. Further details of verification and validation are presented in (Human 2019).

### Simulation Experiments

In this article, 2 case studies that focus specifically on refuge size and layout specifications are presented. These case studies can be used to provide general recommendations for real-world refugia planning in Bt sugarcane in South Africa. The 2 case studies are as follows: (i) finding the right size and distribution of refuge areas and (ii) finding a suitable shape for refuge areas. In each case study the parameter values as given in Table 1 were used. Parameters that remained constant in all experiments are labeled as non-variable. The resistance trait was set as recessive and the Bt crop was set to 2 independent MOA. The Bt protein was set to have a 95% efficacy per MOA, making it 99.75% effective against agents with no resistance to either MOA. The proportion of initial resistant insects was 0.5%.

**Table 1.** The parameters and their corresponding values and units used in each simulation run for the different case studies are presented. Parameters that remained constant in all experiments are labeled as non-variable. The default values assumed for variable parameters in the case study analysis are shown in bold

Object	Parameter	Value(s)	Unit	Variable
Field	Harvest cycle	24	Months	Yes
	Bt modes of action	2	N/A	Yes
	Bt efficacy	95	Percent	Yes
	Gene inheritance	Recessive	N/A	Yes
Eggs	Initial population	0	Agents	No
	Mortality (daily)	3	Percent	No
Larvae/pupae	Initial population	0	Agents	No
	Mortality (outside)	90	Percent	No
	Mortality (inside)	10	Percent	No
Female moth	Initial population	500	Agents	No
	Female likelihood to move 1 sky cell away $\left(m_{s}^{1}\right)$	{9, **18**, 40}	Percent	Yes
	Female likelihood to move 2 sky cells away $\left(m_{s}^{2}\right)$	{1,**2**,10}	Percent	Yes
	Female likelihood to move to neighboring cells $\left(m_{f\;}\right)$	{10, **20**, 30}	Percent	Yes
	Mortality	20	Percent	No
	Ground cell movement	Biased random	N/A	Yes
	Proportion susceptible	99.5	Percent	Yes
	Proportion heterozygotes	0	Percent	Yes
	Proportion resistant	0.5	Percent	Yes
Male moth	Initial population	500	Agents	No
	Male likelihood to move to neighboring cells $\left(m_{m}\right)$	{10, **20**, 30}	Percent	Yes
	Mortality	20	Percent	No
	Sky cell movement	Biased random	N/A	Yes
	Proportion susceptible	99.5	Percent	Yes
	Proportion heterozygotes	0	Percent	Yes
	Proportion resistant	0.5	Percent	Yes

The model was run for 10 yr, and to eliminate the worst initialization bias, the first 4 yr, or 1,460 days, were considered a warm-up period to account for some significant population spikes that occur as the population starts to grow. Both Bt sugarcane and resistance genes are present during the warmup, and results where Bt is only explicitly introduced only after the conclusion of the warmup period is described in detail in Human (2019). Results were only derived from the remaining 6 yr. Each parameter combination tested was run 15 times to account for the inherent stochasticity of a simulation model. The coefficient of variation for control runs with no resistance is less than 0.05, so for a confidence interval width of 0.05, only 7 simulation runs were required (Byrne 2013). The mean and standard deviation used was based on the average number of adult moths in a run. More runs were added to account for the added variability when resistance development is included, hence 15 simulation runs per parameter combination tested were run. To ensure that moths were able to reach any part of the simulation space, the starting conditions for the warm-up period were set, instead of having a single point starting population, to having an evenly distributed population, with 20 evenly distributed batches of 1,000 adult moths inserted across the simulation domain. There were thus a total of 20,000 moths in the simulation at the start of the warm-up period, with 100 being fully resistant. Simulation run duration varied from between minutes to 6 h, directly proportional to the number of agents in the system. For example, runs containing many resistant moths would result in an overall larger population, consistently slowing down the time to complete each iteration.

The parameters and initial values for the case studies may not be reflective of a realistic scenario as there is very little data to work with. For this reason all scenarios developed were designed to be pessimistic. This does lead to results that are more conservative, but it also makes them more interesting, as overly optimistic scenarios were found to be uninteresting, specifically where initial resistance was very low.

### Case Study 1—Refugia Distribution

**Fig. 3.**
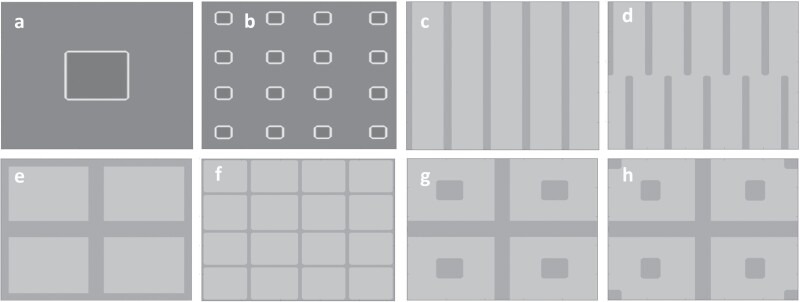
The refuge types considered in the simulation experiments are illustrated. The distributed refuge block layout considered in the first case study, expands in a fractal manner, starting with a (a) single block of refuge and expanding into 4 and then (b) 16 blocks, increasing the distribution, but not the overall size, of the refugia. The (c) linear block 1, (d) linear block 2, (e) border 1, (f) border 2, (g) border and block, and (h) revised border and block refuge layouts, all with h=20%, that are considered for the second case study where the effectiveness of different refuge shapes are investigated. The light gray represents Bt sugarcane, whereas the dark gray represents refugia.

In the first case study, the focus was on the distribution of refugia in a given area. In this scenario, given a proportion of refugia, h∈{10%, 20%, 30%}, 3 layouts were tested. The h values were chosen to be less than or equal to 30% as a higher proportion of refugia is expected to result in significant push-back from growers. The first layout used is a single large block in the center of the simulation space, the second divides the simulation space into equal sized quadrants and places a quarter of the original layout’s refuge in the center of each quadrant, resulting in 4 equal sized refuge areas. The third layout applies the same transformation to the quadrants of the second layout, resulting in 16 refuge areas. Visual representations of the refugia layouts and their fractal expansion may be seen in Fig. 3a–b.

The key outcome of this case study was to evaluate whether or not having more distributed (or scattered) refuge areas would help curb development of resistance. The second key outcome, was to determine whether or not, for any distribution, enough refugia has been allocated to help curb resistance development. In these scenarios, having h=10% split over 16 blocks of refuge may not perform well due to the individual refuge areas being too small to support a significant susceptible insect population. For larger total refuge areas, it is expected that more refuge areas should provide better protection against resistance development, as the average distance between any point in the Bt sugarcane to a refuge area will be smaller as the number of refuge areas is increased.

### Case Study 2—Refuge Shapes

This case study focused on linear block (or bracket) and border strategies. Strip planting was considered infeasible in current sugarcane planting practices, and does not form part of the scope of this study. For the linear blocks, 2 different layouts, as seen in Fig. 3c and d, were tested for the median h value of 20%. The rationale behind the layout in Fig. 3d is that the more distributed refuge areas may allow for more overlapping of susceptible populations, especially on the edges of the simulated space, an area identified as particularly vulnerable in the first case study. For the border, the simulation space is first divided into 4 equal squares as may be seen in Fig. 3e, and then into 16 squares as in Fig. 3f. Two block and border combinations were also considered, as shown in Fig. 3g and h.

### Method of Analysis

A number of key outputs were identified for this study for each parameter combination tested. For each combination tested, 15 simulation runs were performed. Key outputs defined are the average population sizes for all types of agents, the maximum population sizes for all types of agents, the number of runs that developed resistance η, the average size of the resistant population $\mu_{r}$, and where resistant populations were focused. The number of runs that developed resistance is an important metric that gives an indication of how successful a scenario was in curbing the development of resistance. For the purposes of the study, a run that developed resistance is defined as a run that, in the last 6 mo, had at least 1,000 fully resistant individual adult moths. This was chosen as the threshold as it was found to be a good indicator of future resistance development – all runs with 1,000 resistant moths developed large resistant populations. The combination of η and $\mu_{r}$ provides an indication of how likely it is for resistance to develop in a given scenario. Two scenarios that develop resistance the same number of times can be differentiated by the severity of the developed resistance, as a high $\mu_{r}$indicates that there was a consistently large resistant population. It is also important to consider these resistance measurements when observing variations in the model outputs, as a scenario with more resistance should drastically increase the number of agents in the model.

## Results

**Fig. 4.**
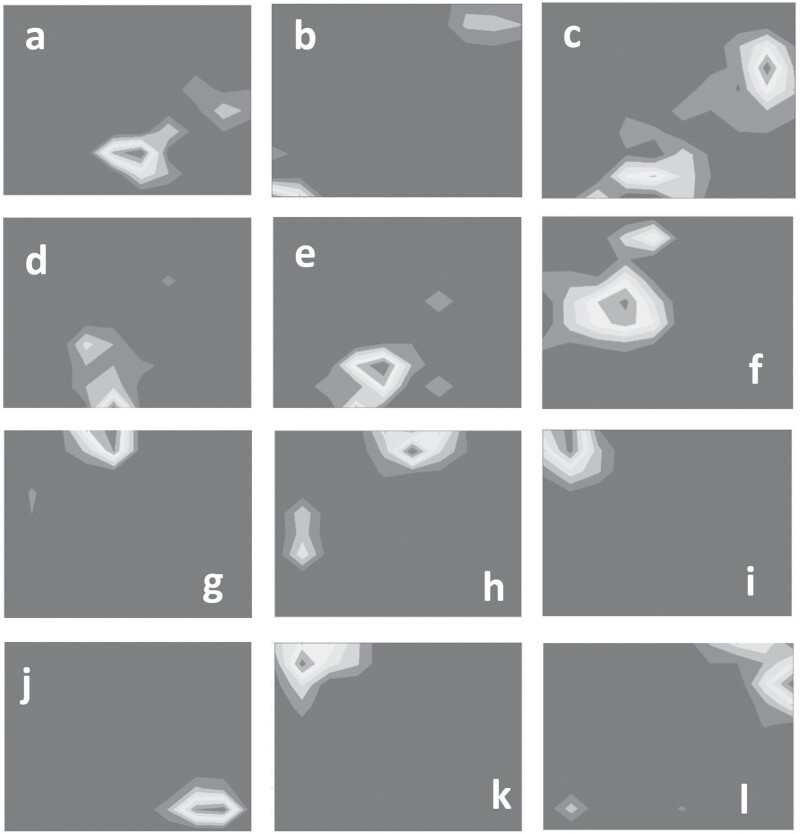
Examples of the concentrations of resistant insects in the sky cell layer at the first point in time of resistance development for the different simulation experiments—with lighter areas indicating higher concentrations. For the experiment where the total refuge size was set to 20% and distributed over 4 blocks across the simulation domain, resistance typically starts developing on the edge of the simulated space, between 2 blocks of refuge, as seen in images (a), (c)–(e). Only in the run shown in the image (b) did resistance develop in a corner of the space. For the experiment where the total refuge size was set to 30% and distributed over 4 blocks across the simulation domain, resistance appears to be focused on the edge of the simulated space, as seen in images (g) and (h). The only case where resistance development seems to have happened more toward the center of the space is the run seen in image (f). Images (i) and (j) represent the 2 border and block refuge runs, whereas images (j)–(l) represent the results of the revised border and block refuge runs. In images (i)–(l), the total refuge size was set to 20% and movement likelihood was set to high. Resistance appears to be focused on the edge of the simulated space.

The most noticeable and arguably most intuitive trend, as may be seen in Fig. 4a, is that a higher proportion of refuge (*h*) value is correlated with fewer cases of resistance development. However, there does not seem to be such an obvious trend in the different layouts, with some results being particularly counter-intuitive. For h=10%, the best result is observed where there are 4 blocks of refuge, but the exact opposite is observed in the cases where h=20%, and h=30%. When excluding h=10%, considering the results in Fig. 4a as a whole, it would appear that either having a single, large refuge area or many smaller refuge areas would be most effective. Although the number of runs that developed resistance η=3 for h=20% distributed over 16 blocks, upon further investigation it was found that 2 of the 3 resistant runs were barely within the threshold for resistance, as both had a maximum resistant population of about 2,000 moths. For the case where h=10%, individual refuge areas are too small in the case of 16 blocks (<1% of total area) to maintain a significant susceptible population. This result also aligns with that of Caprio (2001) that found that source-sink dynamics in fine-grained refuge areas lead to more rapid resistance development than in scenarios with larger, more congruous refuge areas. For h≥20%, the large, single blocks of refuge reduce the need and the likelihood of the moths moving into Bt sugarcane, resulting in it being an effective strategy.

**Fig. 5.**
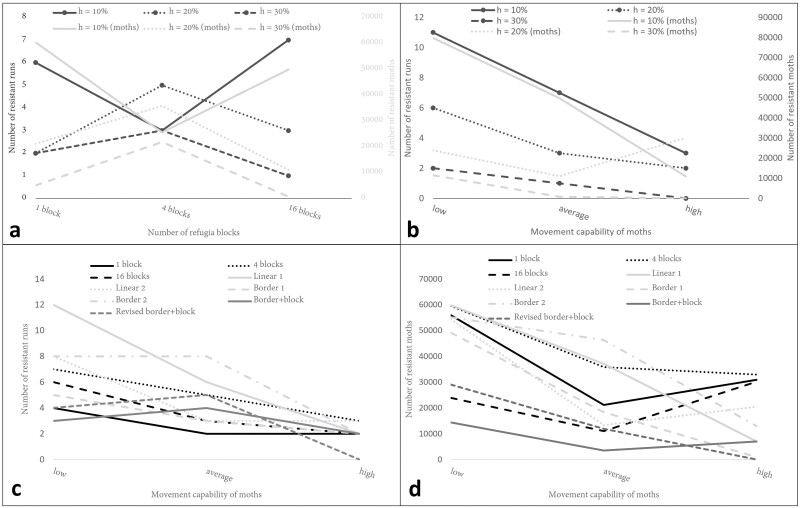
(a) The number of runs η that develop resistance, and the corresponding average number of resistant moths $\mu_{r}$ at the end of 10 simulation years, out of 15 simulation runs per combination of total refuge size h and different distributions across the spatial domain. In image (a), moths were assumed to have average movement capability, with movement likelihood set at the default movement parameter values. (b) The number of runs  η that develop resistance, and the corresponding average number of resistant moths $\mu_{r}$ at the end of 10 simulation years, out of 15 simulation runs per combination of total refuge size, h, and movement capability of the moths. The total refuge area h across the spatial domain was set to be distributed across 16 smaller blocks in all simulation runs. (c) The number of runs η that develop resistance, out of 15 simulation runs per combination of refuge shape or distribution, and movement capability of the moths. The total refuge size h was set to 20% in all simulation runs. (d) The average number of resistant moths $\mu_{r}$ at the end of 10 simulation years, out of 15 simulation runs per combination of refuge shape or distribution, and movement capability of the moths. The total refuge size h was set to 20% in all simulation runs.

To determine what led to the results obtained for the 4 blocks of refuge, it is best to consider where resistance first developed. For this investigation, the grid of sky cells is considered at the earliest point in time that there were more than 1,000 resistant moths and plotting where they are concentrated. In Fig. 4a–e, the locations of initial resistance development may be seen. It does appear that there is a preference for resistance to start developing on the edge of the simulated space, between the 2 blocks of refuge, as seen in Fig. 4a, c, d, and e. Only in the run shown in Fig. 5b did resistance develop in a corner of the space. For h=30% only 3 runs developed resistance as may be seen in Fig. 4f–h. As with the case where h=20%, the development of resistance appears to be focused on the edge of the simulated space, as may be seen in Fig. 4g and h. The only case where resistance development seems to have happened more towards the center of the space is the run seen in Fig. 4f. From these results, it would appear that, for the case where there are 4 blocks of refuge, the increased number of moths that can migrate towards the edge of the simulated space allows for greater survival of the resistance gene. Given enough time, the clusters of moths carrying the resistance gene that do make it to the edges would eventually be in high enough concentrations to create large populations of resistant insects. The same is not observed for the single block cases, as there is too great a distance between the edge of the refuge and the edge of the simulation space, making it far less likely for moths to get “stuck” on the edges. Where there are 16 blocks of refuge, the refuge areas are close enough to the edge for the susceptible and resistant insects to interact regularly, leading to less resistance development.

The best results obtained from the average movement cases were for h=30% and 16 blocks of refuge. To stress test these results, the experiments are repeated for the high and low movement scenarios for all values of h. For these tests, 2 sets of experiments were run. The first set compares the different movement parameters to the 16 blocks of refuge cases forh∈{10%, 20%, 30%}, while the second compares the movement parameters to the 1, 4, and 16 blocks cases where h=20%, which was the median case of the previous results. The combination of these experiments should indicate how resistance development correlates with both moth movement and refuge size and distribution.

From Fig. 5b, it may be seen that for all 3 h values, the low movement cases were much more prone to resistance development. This indicates that, where the moths are not likely to move very far, there is a point where even highly distributed refugia cannot guarantee that susceptible and resistant insects will ever meet, and resistance can develop quickly. In general, where movement parameters were very high, resistance did not develop as easily, hence there is a correlation between likelihood of movement and likelihood of resistance development. Where h=20% in the high movement case, 1 run spontaneously developed resistance near the end of the 10 yr run (including warm-up), which resulted in it not being better than the average case. This single run of resistance development begged the question of how rapidly resistance would spread through a population once it starts gaining momentum.

In Fig. 5c and d, for the 1, 4, and 16 block refugia distributions, it may be seen that lower movement led to resistance development more often, while there is a low ratio of resistance development in the higher movement case. However, there is not a very clear distinction on which of the 1 or 16 block layout performed better, except that the results for a single large block did appear to be slightly better at curbing resistance when looking at the number of runs that developed resistance. This result does coincide with those obtained in Fig. 5a where it was stated that a very large or many smaller refuge areas are the most effective.

**Fig. 6.**
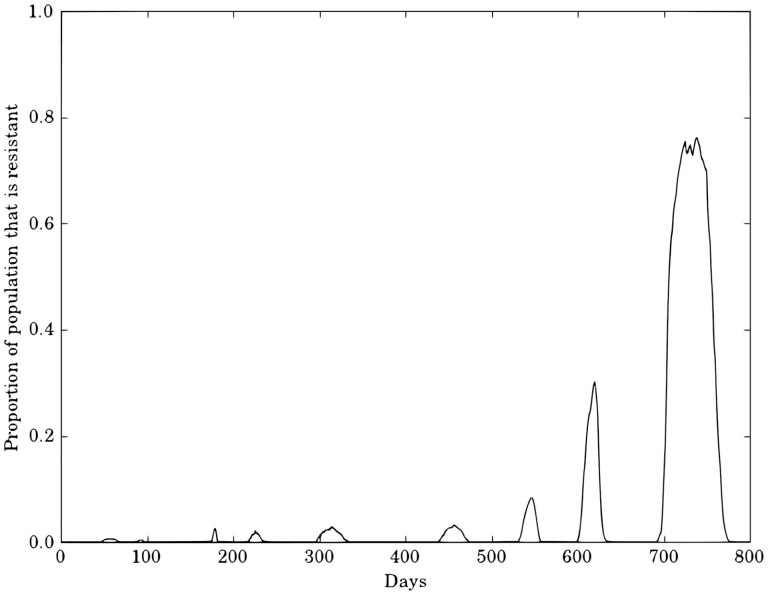
Example simulation output for a run in the high movement case with total refuge area h=20% distributed across 4 blocks over the simulation domain. The time from having less than 1% resistance in the entire population to having over 75% resistance was less than 800 days.

All experiments in the case studies started with exactly 100 resistant moths, and in runs where resistance did not develop, 100 remains the maximum number of resistant moths throughout the run. For all the runs that developed resistance, it was found that from the point where the resistant population goes over 100, to the point where it reaches 100,000 took an average of 709 days, or approximately 1 harvest cycle. In Fig. 6, it may be seen that, for a run in the high movement case with h=20% and 4 blocks of refuge, the time from having less than 1% resistance in the entire population to having over 75% resistance was less than 800 days.

As the initial 100 resistant moths are evenly distributed among the 20 starting populations, it is likely that there exists a critical point. This point is where either the number or concentration of resistant moths in the same area becomes large enough that resistance becomes inevitable, and can be approximated by taking the maximum number of resistant moths that appear in a sky cell in all models that do not develop resistance, as well as the highest ratio of resistant to susceptible insects in a sky cell. Taking the results from the previous experiment where no resistance was observed, with h=30% and high movement, the maximum number of resistant moths in a sky cell was only 13. The maximum ratio of resistant to susceptible moths, where there are at least 50 total moths in a sky cell, was approximately 1 to 9. When considering the high movement case where h=20%, if the first point in time after initialization is considered where the total number of resistant insects reach 100 for runs that did develop resistance, the maximum ratio of resistant to susceptible insects was 1–5. For the resistant runs from h=10%, and high movement, this ratio was 1–4 resistant to susceptible insects. From this, it can be assumed that the critical point/ratio would then lie between 1:4 and 1:9 resistant to susceptible insects in a single sky cell.

As observed in the first case study, the general trend seems to be that more mobile insects led to fewer cases of resistance developing across all different refuge layouts. As may be seen in Fig. 5c and d, the Linear 1 and Border 2 or arguably the worst performing layouts. Staggering the linear blocks as in Linear 2 does appear to have the desired effect, with the cases of low and average movement outperforming those of Linear 1 and the case for high movement only being slightly worse. The layout that nearly dominates all other linear and border layouts is Border 1, with the lowest number of runs that developed resistance (*η*) values for all movements and a lowest average size of the resistant population ($\mu_{r}$) for 2 of the 3 movements. When compared to the case where h=20% for 1 and 16 blocks, Border 1 compared favorably, having η and $\mu_{r}$ values that fall in between the block refuge results.

The fact that Border 2 performs worse than Border 1 again speaks to the third point made by Ostlie et al. (1997), where it is stated that refuge areas should (i) be able to sustain susceptible insects and (ii) should allow for susceptible insects to come into contact with resistant ones. For Border 2, the refuge areas are much more distributed, which does agree with the second point, but the refuge areas are only a single ground cell wide, which may not be large enough for the first point to hold. It is also interesting to note that for the high movement cases, the shape of the refuge made very little difference, supporting the result that, where insect dispersal is high, only the size of the refuge has a significant impact. This result supports the model implementations of Sisterson et al. (2005) and Peck et al. (1999) where only refuge size was considered, as the target pests were considered to disperse over large areas.

Since, for h=20%, block and border refuge performed the best so far, it was decided to experiment with combining the 2 layouts. In Border 1, the borders were only 1 ground cell wide and this was found to be insufficient for maintaining a healthy susceptible population. For this experiment, the cells making up the border of Border 2 are aggregated into blocks of refugia as may be seen in Fig. 3g and h. In the first case study, it was found that resistance was most likely to develop towards the middle of the edges. With the combination refuge, there are now refuge areas extending into those edges that can help curb the development of resistance. The border and block combination layout does appear to be effective in preventing the development of resistance across all movements. The η and $\mu_{r}$ values in Fig. 5c and d are mostly improved when compared to those achieved by the Border 1 test or the block refuge test where h=20%. This indicates that 2 different refuge structures with distinct weak spots can be combined to form a more effective hybrid. The 4 block refuge had weaknesses at the center of the edges and Border 1 had points in the middle of the Bt crops that were very far from refuge. In the hybrid, the border portion provided refuge near the edges, addressing the 4 block weakness, and the 4 blocks covered the vulnerable centers of the Border 1 layout.

With the edges and the vulnerable centers covered, it was hypothesized that resistance would develop in the corners of the simulated space. To investigate, the 2 runs from the high movement case where resistance developed were considered, as it was assumed that these runs would highlight the biggest flaws in the refuge strategy. In Fig. 4i and j it may be seen that this is indeed the case, with both initial resistant populations tending towards the corners of the simulated space. To see if placing refuge areas in the corners would counteract this result, the original experiment was revised to include refuge areas in the corners as may be seen in Fig. 3h. For the high movement scenario, as may be seen in Fig. 5c and d, there was no resistance development at all. Unfortunately, in both the low and average movement scenarios the revised layout performed worse than the initial layout. Taking a sample of 3 runs that developed resistance, the points of initial resistance development may be seen in Fig. 4j–l. The resistance seems to develop just off of the edges of the corner refuge areas, indicating that these refuges may simply be too small to have a real impact in low or average movement scenarios.

## Discussion

Given the large number of variables in the model, it was infeasible to run simulations for all possible combinations of variable values. However, the results presented in this paper should still allow for making high-level recommendations of how to approach refugia planning in a Bt sugarcane scenario with respect to *E. saccharina*. For a successful refuge strategy, there are some key basic assumptions that need to hold about the Bt sugarcane and the target pest, *E. saccharina*, before a refuge planting strategy can even be considered. The results obtained in this study provide further evidence of the validity of assumptions presented by Ostlie et al. (1997). The 4 key assumptions identified in our research are: (i) *High dose, high efficacy*: The development of resistance was best curbed where the efficacy of the Bt protein was at least 85%. Many of the most successful scenarios tested were very effective at quickly killing as many moths as possible, hence a very high dose Bt sugarcane would more likely result in a delay in resistance development. (ii) *Multiple modes of action*: One mode of action was found to be highly susceptible to resistance development. The default in this study was 2 modes of action, but more modes of action are necessarily better. (iii) *Recessive resistance genes*: In a preliminary scenario that was tested, where the genes for resistance were dominant, even with 2 modes of action resistance developed readily. For the successful introduction of Bt sugarcane, the genes conferring resistance must be nearly or completely recessive. (iv) *Low initial gene frequency*: Most scenarios in this study had a relatively high initial resistance gene frequency in an effort to stress test the scenario set-up. In reality, the gene should be sufficiently rare (≤0.1%) to successfully curb resistance. In tests where the starting resistant population was 0.1%, resistance never developed. This is arguably the most important assumption as having too many insects initially resistant would most likely make any realistic refuge strategy fail.

If the 4 basic assumptions hold, refugia planning may be considered. Based on the results obtained from the case studies presented, the maximum proportion of a planted field to be kept as refuge is set at h=30%, which is slightly higher than recommendations found in the literature. This can be used as the conservative estimate for the refuge required in nearly any scenario, including low or high mobility. The recommended minimum is set at h=10%, although this should only be considered if there is high confidence that the resistance gene is very rare (≤0.1%), and if *E. saccharina* displays high movement capability. This corresponds with results obtained by Cristofoletti et al. (2018), who recommended a refuge size of 10−20%.

The experiments considered in the 2 case studies provided evidence that not all refuge shapes are equally effective. Block refuges were found to be very effective in most cases and may potentially be improved upon by adding an element of the border refuge approach, but the difference was not significant enough to justify such a complex structure. The linear structures did not perform very well overall and may not warrant any further consideration. When considering the distribution of refuge areas, having large refuge areas may be more effective at curbing resistance development than having many small ones, especially if the infestation tends to be more localized (low movement). This correlates strongly with the results obtained by Tyutyunov et al. (2008), where for small to medium farms it was also found that fewer, contiguous refuge areas were more effective. If a population of moths exists within a large refuge area, the surrounding Bt would act as a wall, reflecting the population towards the center of the refuge area. If the refuge area is sufficiently large enough, the moths will not have any reason to leave the refuge area, reducing the risk of resistance development.

A key indicator of how successful a refuge strategy was, was what movement assumption was made about the moth. Moth populations that tend to be very localized would benefit from a more conservative refuge strategy, whereas moths that were more likely to disperse over greater distances allow for a more lenient refuge requirement. The layout of the refuge is also less important where moths tend to disperse over greater distances, and most strategies performed nearly equally well when high moth movement was considered. If the moth is assumed to be a low movement species, the best strategies have larger individual refuge areas and it is recommended that a layout such as a central, single block, or Border 1 be considered.

Of course, any simulation model developed contains several simplifying assumptions and, as such, cannot be considered a complete and accurate representation of the real-world interactions between *E. saccharina* and sugarcane, but rather as an approximation. Most of the assumptions made when setting parameter values were supported by past research of *E. saccharina*’s general biology and how it infests sugarcane. Some behaviors of the moth were implemented as a “best guess” estimate and presented to subject matter experts to evaluate. Future research into these behaviors in particular would greatly benefit the development of improved models. For example, there is a lack of experimental data related to how fast and how far the *E. saccharina* moth disperses once it emerges. It was assumed that the female was able to disperse further and more readily than the male and that males tend to move more towards more mature sugarcane to form *leks* based on observation by experts. While it could be realistically assumed that there is no outward difference between a normal and a Bt sugarcane varietal, it is not currently known whether or not *E. saccharina* will display some notable bias when choosing an egg laying site. It is currently assumed that the female is agnostic and would equally likely choose an egg laying site, but future research into any potential bias would allow for a more realistic future model.

In the scenarios tested in this study, it was assumed that there are no other control strategies in place other than the use of Bt sugarcane. In reality, it is much more likely that the grower would incorporate a variety of control strategies and the inclusion of these strategies in a future model could assist in finding good combinations for pest management. For example, Tabashnik et al. (2010) discussed the efficacy of combining sterile insect releases with the usage of Bt cotton where there were infestations of pink bollworm (*Pectinophora gossypiella*) and found that refugia became redundant.

The case studies presented in this paper, highlighted that not all refuge areas are equally effective, however, most refuge recommendations presented in the literature are only focused on the overall size of the refuge area. Having highly distributed refuge areas may mean that individual refuge areas may not be large enough to sustain a sufficiently large susceptible population. It was also found that there is a significant difference between different layouts, even if they were overall the same size. Another key outcome is the effect of dispersal rates on how effective refuge strategies were. It was found that there was an inverse relationship between the insects' likelihood of movement (dispersal rate) and resistance development.

Results indicate a number of edge effects, mainly due to the reflective hard boundary that was implemented. While sugarcane is grown in large expanses in South Africa, there are many areas which are not contiguous, especially smaller farms, that are surrounded by wetlands or other non-sugarcane areas. In these cases, edge effects should be accounted for. Ideally, an inlet-outlet boundary would be preferred, but would require rigorous assumption setting about movement across the boundary. A toroidal layout can also be used, to remove explicit assumption setting about boundary conditions, but with its own implicit assumptions. A toroidal layout assumes that the number and genetic composition of the agents leaving the one end of the simulation space and entering the other end remains the same.

As a risk management tool, the model can be expanded to an area covering several square kilometers. This would align the model with the regional models developed by Sisterson et al. (2004, 2005)Peck et al. (1999), Caprio and Tabashnik (1992), and Caprio (1998). Given the results that not all refuge areas are equally effective, a further aspect to consider, is that sugarcane farms exist in large expanses where there are many different layouts and structures such as roads and other unplanted areas. In addition, commercial growers can be located adjacent to small-scale growers, and may not follow the same farming practices. Reading in distinct geographic information system (GIS) shapefiles would allow for customized refuge recommendations according to the layout of a specific sugarcane region. This paper did not consider an economical or behavioral analysis. Social factors have been shown to have a substantial impact on insect resistance development, and should be taken into account in policy analysis studies (Saikai et al. 2020). A risk-benefit analysis and overall financial impact of non-compliance should be considered, especially in cultures where people tend to make individualistic decisions for personal short-term gain.

As a final comment, many simulation models are designed to investigate what might happen to a system given a certain set of input parameters. In this study, the aim was to provide general recommendations on refugia planning using several parameter variations and scenarios, supporting the case by Saikai et al. (2020) for using agent-based simulation models to aid explicitly in decision support for policy evaluation. Given what is currently unknown about *E. saccharina* in terms of resistant gene frequency and movement capability, and the simulation results presented in this paper, we recommend a conservative initial policy of large block refuges, with a refuge size of 30% on any specific farm.
